# Domesticated cynomolgus monkey embryonic stem cells allow the generation of neonatal interspecies chimeric pigs

**DOI:** 10.1007/s13238-019-00676-8

**Published:** 2019-11-28

**Authors:** Rui Fu, Dawei Yu, Jilong Ren, Chongyang Li, Jing Wang, Guihai Feng, Xuepeng Wang, Haifeng Wan, Tianda Li, Libin Wang, Ying Zhang, Tang Hai, Wei Li, Qi Zhou

**Affiliations:** 1 State Key Laboratory of Stem Cell and Reproductive Biology, Institute of Zoology, Chinese Academy of Sciences, Beijing, 100101 China; 2 Institute for Stem Cell and Regeneration, Chinese Academy of Sciences, Beijing, 100101 China; 3 University of Chinese Academy of Sciences, Beijing, 100049 China

**Keywords:** embryonic stem cells, blastocyst complementation, cynomolgus monkey, pig, interspecies chimera, organ reconstruction

## Abstract

Blastocyst complementation by pluripotent stem cell (PSC) injection is believed to be the most promising method to generate xenogeneic organs. However, ethical issues prevent the study of human chimeras in the late embryonic stage of development. Primate embryonic stem cells (ESCs), which have similar pluripotency to human ESCs, are a good model for studying interspecies chimerism and organ generation. However, whether primate ESCs can be used in xenogenous grafts remains unclear. In this study, we evaluated the chimeric ability of cynomolgus monkey (*Macaca fascicularis*) ESCs (cmESCs) in pigs, which are excellent hosts because of their many similarities to humans. We report an optimized culture medium that enhanced the anti-apoptotic ability of cmESCs and improved the development of chimeric embryos, in which domesticated cmESCs (D-ESCs) injected into pig blastocysts differentiated into cells of all three germ layers. In addition, we obtained two neonatal interspecies chimeras, in which we observed tissue-specific D-ESC differentiation. Taken together, the results demonstrate the capability of D-ESCs to integrate and differentiate into functional cells in a porcine model, with a chimeric ratio of 0.001–0.0001 in different neonate tissues. We believe this work will facilitate future developments in xenogeneic organogenesis, bringing us one step closer to producing tissue-specific functional cells and organs in a large animal model through interspecies blastocyst complementation.

**Electronic supplementary material:**

The online version of this article (10.1007/s13238-019-00676-8) contains supplementary material, which is available to authorized users.

## Introduction

Human pluripotent stem cells (hPSCs) have the ability to self-renew and generate a large variety of cell types, and may be capable of generating human organs in other mammals for future organ transplantation by xenogenesis (Wu et al., 2016). Previous studies have demonstrated the feasibility of generating an entire organ by xenogenesis, using rodent PSC blastocyst complementation. Hiromitsu et al. used rat PSCs to complement pancreatic and duodenal homeobox 1 (*Pdx1*)-deficient murine blastocysts, and generated an entire rat pancreas with normal physiological function inside a *Pdx1*-null murine host (Kobayashi et al., 2010). A similar study reconstituted a rat thymus in a nude mouse (Isotani et al., 2011).

However, xenogenesis is more complex in humans than in mice because hPSCs exist in a “primed” state (Rossant, 2015). Primed hPSCs, which show some similarities to mouse epiblast stem cells (EpiSCs) (Nichols and Smith, 2009), can participate in normal mouse development when transplanted into gastrula-stage embryos. Region-selective hPSCs in a primed state could also generate post-implantation interspecies chimeric embryos after grafting to the posterior epiblast of gastrula-stage mouse embryos (Wu et al., 2015). Although primed hPSCs could not participate in normal mouse development when transplanted into preimplantation embryos as naïve PSCs (Mascetti and Pedersen, 2016), inhibiting apoptosis was shown to enhance the chimeric ability of primed hPSCs in mice (Huang et al., 2018; Wang et al., 2018b). In addition, Kang et al. showed that primed PSCs from cynomolgus monkeys (Macaca fascicularis) could generate chimeras using optimal culture conditions (Kang et al., 2018). Several recent studies have also reported the generation of naive PSCs, which showed higher efficiency in single-cell cloning and genome editing, and could be incorporated in the host when transplanted into preimplantation embryos (Gafni et al., 2013; Chen et al., 2015). Moreover, an intermediate primed state, between the naïve and primed state, was proven to contribute to the generation of chimeras by blastocyst injection (Tsukiyama and Ohinata, 2014), and resulted in better developmental ability of a chimeric pig (Wu et al., 2017).

Presently, mouse models are primarily used as chimeric hosts. However, these models are not suitable for translational chimeric research because of the marked differences in development (i.e., embryo size and gestational period) between rodents and humans. Izpisua et al. pioneered chimeric research in a large animal model, successfully incorporating a xenogenous graft of hPSCs into pig blastocysts and confirming hPSC survival in early stage porcine embryos (Wu et al., 2017). However, ethical issues prevent the study of human chimeras in the late stage of embryonic development, and it is unknown whether human cells can contribute to the organs of chimeras. Primate PSCs, which have similar pluripotency to hPSCs, are a good model for studying interspecies chimerism and organ generation.

In this study, we used cmESCs to explore the possibility of interspecies chimerism in the development of late embryonic stage pigs. By optimizing the medium in which the cmESCs and injected blastocysts were cultured, we observed an increase in the anti-apoptotic ability of cmESCs and in the extent of chimeric embryo development, resulting in the successful incorporation of xenogenous cmESC grafts into multiple tissues of the neonatal pigs. This work will enable developments in xenogeneic organogenesis towards producing tissue-specific functional cells and organs in large animal models via interspecies blastocyst complementation.

## Results

### Chimeric competency of cmESCs in different pluripotent states

**Figure 1:**
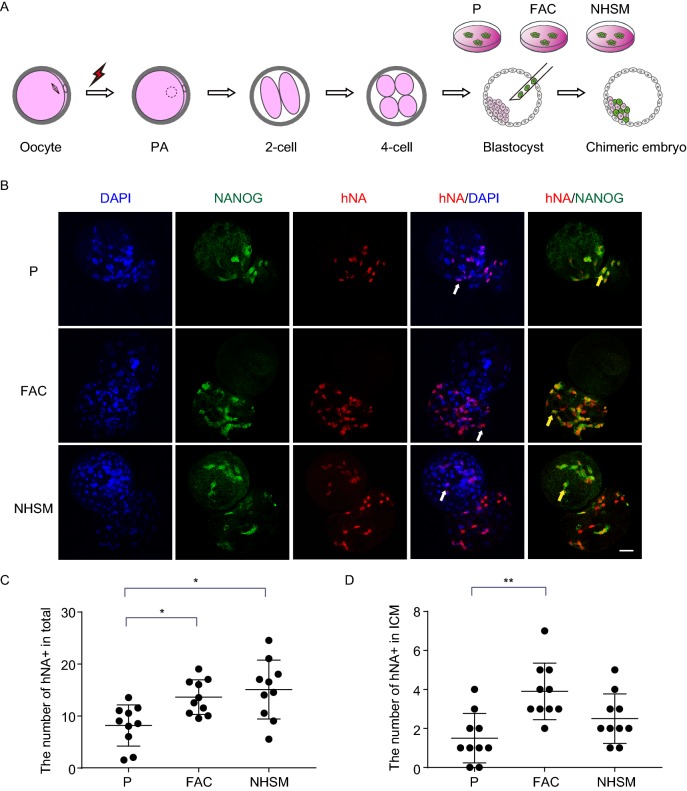
**Chimeric competency of cmESCs in different pluripotent states**. (A) Experimental procedures used to evaluate the chimeric competency of cmESCs in three pluripotent states by blastocyst injection. (B) Representative images of porcine PA embryos showing integrated hNA positivity, expression of NANOG (an ICM marker), and DAPI staining (a marker of total cells) in cultured chimeric embryos. White arrows, hNA positive/DAPI cells; yellow arrows, hNA positive/NANOG positive cells; scale bars, 50 µm. (C) Number of cmESCs in different pluripotent states integrated into the total porcine blastocyst. (D) Number of cmESCs in different pluripotent states integrated into the porcine ICM. *P < 0.05; **P < 0.01, Student’s *t*-test. Error bars represent the mean ± SEM (*n* = 10)

To systematically evaluate the chimeric competency of cmESCs in pigs, we prepared cmESCs in three pluripotent states: primed (P) (Thomson et al., 1998), intermediate (FAC) (Tsukiyama and Ohinata, 2014; Wu et al., 2017), and naïve (NHSM) (Gafni et al., 2013; Chen et al., 2015). Cells in all three states expressed the pluripotency factor POU class 5 homeobox 1 (POU5F1), differentiated into embryoid bodies *in vitro* and teratomas *in vivo*, which contained tissues derived from all three embryonic germ layers, and also showed the normal the karyotypes (Fig. S1A–D). To determine whether the cmESCs could be engrafted into the porcine inner cell mass (ICM), we injected 10 GFP-labeled cmESCs from each pluripotent state into porcine parthenogenetic (PA) blastocysts, followed by 48 h of culture *in vitro*, and then evaluated their chimeric contributions (Fig. 1A). Three criteria were used to evaluate the chimeric contributions of the cmESCs in porcine blastocysts: survival of cmESCs in the embryonic environment (the percentage of GFP-positive embryos divided by the number of embryos), the ability and efficiency of cmESCs to incorporate into total blastocysts (the number of anti-human nuclear antigen (hNA) antibody-positive cells divided by the number of cells), and their ability to incorporate into the ICM (the number of anti-hNA antibody-positive cells among the Nanog homeobox (NANOG)-positive cells). There were no significant differences in cmESC survival between the different states (Fig. S1E and S1F). However, the average numbers of cmESCs integrated into each blastocyst in the intermediate state (27.2 ± 2.107, *P* < 0.05) and naïve state (30.1 ± 3.585, *P* < 0.05) were higher than in the primed state (16.4 ± 2.504). Furthermore, the average number of cmESCs integrated into the ICM in the intermediate state (3.9 ± 0.458) was higher than in the primed states (1.5 ± 0.401, *P* < 0.01) (Fig. 1B–D). These results indicated that *in vitro*, cmESCs in the intermediate pluripotent state had better survival and ICM integration ability in pigs compared with the other cell types.

We next investigated whether cmESCs in the different states contributed to neonatal porcine development *in vivo*. In total, 1,203 blastocysts derived from chimeric embryos were transferred to surrogate sows after 24 h of *in vitro* culture. Surprisingly, no natal chimeras survived, regardless of the culture system (Table S1). We suspected that 24 h of *in vitro* culture resulted in fatal damage to embryonic development, especially among the *in vitro* embryos, whose quality may have been worse than that of embryos fertilized *in vivo*, making them more sensitive to external factors.

### Domesticated ESC medium positively affects both ESCs and embryos during chimeric generation

Since *in vitro* culture of chimeric blastocysts appeared to have damaging effects on the embryos, we sought to improve the chimeric system. Previous studies have demonstrated that overexpression of anti-apoptotic genes significantly improves the chimeric ability of human ESCs in mice. We therefore hypothesized that inhibition of apoptosis might enable the cmESCs to form interspecies chimeras upon injection into porcine embryos. To test this, we used a doxycycline-inducible system for transient induction of the human anti-apoptotic gene BCL2 like 1 (*BCL2L1*) (Wang et al., 2018b). The survival of cmESCs overexpressing BCL2L1 was improved compared with control cmESCs, and the average number of BCL2L1-overexpressing cmESCs integrated into each blastocyst *in vitro* was higher as well (Fig. S2A–C). However, we still did not obtain any neonatal chimeras from a total of 643 blastocysts transferred into surrogate sows (Table S1), indicating that other factors influenced interspecies chimera formation.

**Figure 2:**
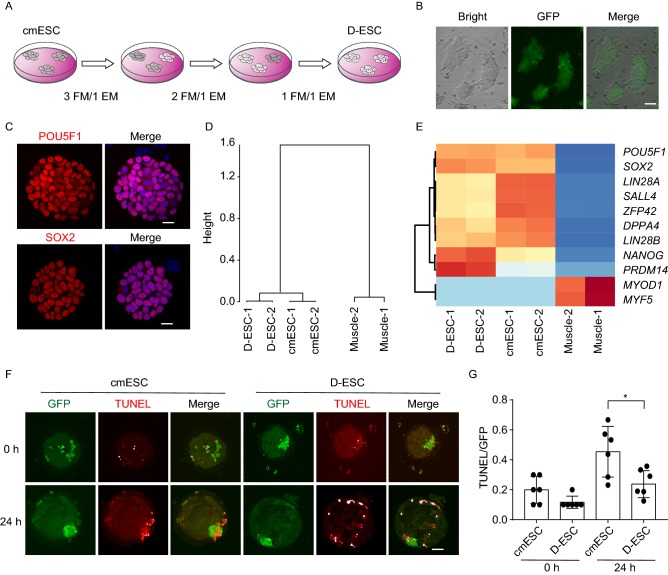
**D-ESC derivation**. (A) The strategy for generating D-ESCs from cmESCs. (B) Representative bright-field, fluorescence and merge images of D-ESC colony morphology. Scale bar, 200 µm. (C) Immunofluorescence images of D-ESCs stained with POU5F1 and SOX2 antibodies. Scale bar, 100 µm. (D) Principal component analysis of gene expression in D-ESCs, cmESCs, and muscle cells. (E) Heat map of pluripotent gene expression in D-ESCs, cmESCs, and muscle cells. (F) Representative fluorescence images of TUNEL-stained porcine PA embryos 0 and 24 h after ESC injection. Scale bars, 50 µm. (G) Proportion of TUNEL-positive/GFP-positive cells 0 and 24 h after ESC injection. *P < 0.05, Student’s *t*-test. Error bars represent the mean ± SEM (*n* = 6)

A comparison of the cell and embryo culture systems showed that the pH and osmotic pressure of the cell culture medium and embryonic medium (EM) differed (data not shown). These differences may have reversed the chimeric process, leading to *in vivo* embryonic development failure after *in vitro* culture. Thus, we improved the cell culture medium to better resemble the EM, by mixing FAC medium (FM) with EM, and changing the FM:EM ratio from 3:1 to 1:1 (Fig. 2A). We named this domestic medium (DM), and termed cmESCs cultured in 1:1 FM:EM domesticated ESCs (D-ESCs), which could be cultured for long periods. They exhibited normal ESC morphology (Fig. 2B) and the karyotypes (Fig. S2D), expressed the pluripotency markers POU5F1 and SRY-box transcription factor 2 (SOX2; Figs. 2C and S2E), and *in vivo*, differentiated into teratomas that contained tissues of all three embryonic germ layers (Fig. S2F). Subsequently, we assessed the transcriptomes of primed cmESCs and D-ESCs using RNA sequencing (RNA-seq). The results revealed no significant differences in RNA expression between D-ESCs and cmESCs. Principal component analysis revealed that D-ESCs displayed the same clusters as cmESCs (Fig. 2D). In addition, naïve markers, such as NANOG and PR/SET domain 14 (PRDM14), were expressed at higher levels in D-ESCs compared to cmESCs (Fig. 2E). To examine the effects of the improved system on chimerism, we first evaluated the anti-apoptotic ability of D-ESCs, and observed enhanced anti-apoptotic ability during chimera development after the 24-h co-culture phase compared with that of cmESCs cultured in FM (Fig. 2F–G). We also evaluated the effects of DM on embryo development. The results revealed similar blastocyst and hatching rates in DM and EM, which were higher than in FM (Table 1). These results suggest that DM is favorable for both ESCs and embryos during chimera development.

**Table 1:** Developmental information of embryo cultured in EM, FM and DM

Groups	No. oocytes	No. blastocysts (%)	No. hatching blastocysts (%)
EM	164	33 (22.10 ± 9.35)^a^	7 (4.72 ± 4.37)^a^
FM	226	11 (5.32 ± 3.04)^b^	1 (0.37 ± 0.64)^b^
DM	189	41 (22.83 ± 5.37)^c^	6 (3.11 ± 0.40)^c^

For statistical comparison, Student’s *t*-test analysis of variance was employed

^a^FM versus EM (*P* < 0.05); ^b^DM versus FM (*P* < 0.05); ^c^DM versus EM (*P* > 0.05)

### D-ESCs can generate interspecies chimeric embryos

**Figure 3:**
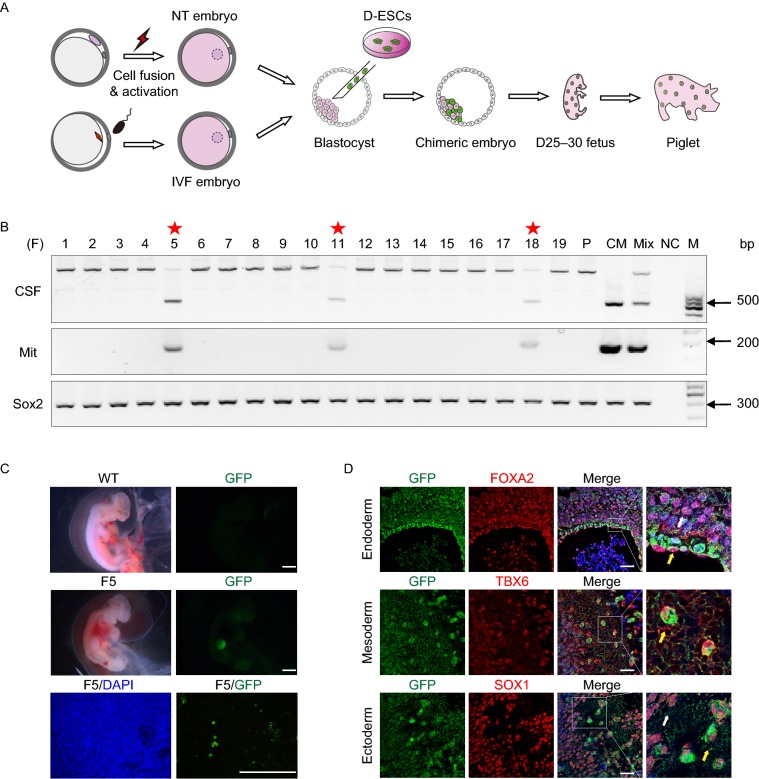
**Generation of post-implantation chimeric embryos**. (A) Schematic of the generation and analyses of post-implantation porcine embryos derived from D-ESC injection into blastocysts. (B) Representative gel images of genomic PCR analyses of D25–D30 porcine embryos using the cynomolgus monkey-specific primers *CSF* and *MIT*. A pig-specific marker, *SOX2*, was used as a loading control. F1–19, fetus samples; P, pig; CM, cmESCs; Mix, pig/cmESCs (1:1); NC, negative control without genomic DNA; red asterisk, positive chimeric sample. (C) Representative bright-field, fluorescence, and immunofluorescence images of GFP-labeled D-ESC derivatives in wild-type (WT) and chimeric (F-5) porcine embryos. Scale bar, 2 mm. (D) Representative immunofluorescence images showing integrated GFP-positive cynomolgus monkey cells and co-expressed lineage markers, including the endoderm marker FOXA2, mesoderm marker TBX6, and ectoderm marker SOX1. White arrows, lineage marker positive cells; yellow arrows, cell positive for both GFP and lineage markers. Scale bar, 50 µm

Next, we investigated the potential contribution of D-ESCs to post-implantation development following transfer to surrogate sows. The embryo manipulation procedures performed are shown in Fig. 3A. In brief, porcine embryos derived through *in vitro* fertilization (IVF) or nuclear transfer (NT) were cultured *in vitro* to the blastocyst stage. Then, 10–15 D-ESCs were injected into each blastocyst, and embryos were collected 25–30 days later for further analysis. Of 4,359 blastocysts transplanted, 59 embryos were obtained, of which three were chimeric. These chimeric embryos collected between 25–30 days were verified by a sensitive genomic polymerase chain reaction (PCR) assay using monkey-specific sequence primers (Fig. 3B). Compared to wild-type (WT) embryos, obvious green fluorescent protein (GFP) expression was observed in the fetus 5 (F5) sample. We verified the GFP-positivity of F5 by immunofluorescence (IF) analysis (Fig. 3C). To determine how D-ESCs were involved in germ layer differentiation, we costained for GFP and various lineage markers. Subsets of GFP-positive cells expressed the endoderm marker forkhead box A2 (FOXA2), mesoderm marker T-box transcription factor 6 (TBX6), and ectoderm marker SRY-box transcription factor 1 (SOX1), suggesting that the D-ESCs could differentiate into all three germ layers (Fig. 3D).

### D-ESCs can generate interspecies chimeric neonatal pigs

**Figure 4:**
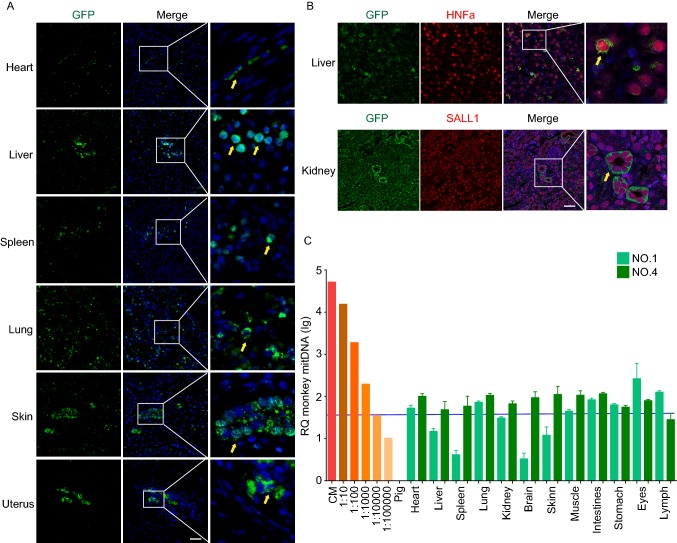
**Chimeric neonatal pigs generated from D-ESCs**. (A) Representative immunofluorescence images of GFP-labeled D-ESCs in the heart, liver, spleen, lung, skin, and uterus of a chimeric neonatal pig. Scale bars, 100 µm. (B) Representative immunofluorescence images showing integrated GFP-positive cynomolgus monkey cells and co-expressed organ markers, including the liver marker HNF4A and the kidney marker SALL1. Yellow arrows, cells positive for both GFP and organ markers. Scale bar, 50 µm. (C) Representative quantitative genomic PCR analysis of cynomolgus monkey mtDNA in the tissues of chimeric neonatal pigs (No. 1 and No. 4) derived from blastocyst injection with D-ESCs. A series of cynomolgus monkey-pig cell dilutions (1:10–1:100,000) were run in parallel to estimate the degree of monkey cell integration (orange bars). Red bar, monkey mtDNA control; green bars, tissue of interspecies chimeras. The dashed line indicates the monkey mtDNA detection level and is equivalent to one monkey cell per 10,000 porcine cells

To evaluate long-term integration, three pregnancies were continued until birth. One of these was spontaneously aborted, in two other pregnancies, 10 pups were born but all died within 1 week. Multiple tissues and organs were collected from neonatal pigs and analyzed for the presence of cynomolgus monkey cells. Two of the pups were chimeras, confirmed by PCR using a monkey-specific sequence primer (Fig. S3A). GFP-positive cynomolgus monkey cells contributed to multiple tissues, including the heart, liver, spleen, lung, and skin, but not to others, including the testis and ovaries, due to low chimerism in the postnatal pigs (Figs. 4A and S3B). To further confirm the fates of the D-ESCs in the neonatal pigs, we co-stained for GFP and tissue-specific markers. Subsets of GFP-positive cells expressed liver and kidney tissue-specific markers, suggesting that these monkey cells were not in a pluripotent state but had differentiated into hepatocyte nuclear factor 4 alpha (HNF4A)-positive liver cells and spalt like transcription factor 1 (SALL1)-positive kidney cells, respectively (Fig. 4B). Through mitochondrial DNA (mtDNA) detection, we found that the chimeric ratio in different tissues ranged from 0.001–0.0001 (Fig. 4C). All data regarding chimeric development with IVF embryos *in vivo* are shown in Table 2. Taken together, these results demonstrated that D-ESCs contributed to all three germ layers and various tissues in the embryonic and neonatal phases, indicating successful interspecies chimerism between cynomolgus monkeys and pigs.

**Table 2:** Developmental and positive efficiencies of D-ESCs after chimeric operation with IVF embryos *in vivo*.

ESC lines	Sex	State	Stage of injection	No. embryo transferred	No. recipient	No. development *in vivo*	
						No. D25–30 embryos GFP+/total	No. full-term GFP+/total
D-ESC-1	Male	Intermediate	Blastocyst	1,620	12	2/33	2/7
D-ESC-2	Male	Intermediate	Blastocyst	1,318	8	1/7	0/3
D-ESC-3	Male	Intermediate	Blastocyst	1,421	9	0/19	0/0

## Discussion

In this study, we injected D-ESCs into porcine blastocysts to obtain neonatal interspecies chimeras. The D-ESCs differentiated into all three germ layers in the pig fetuses, consistent with a previous study reporting human/pig chimeras (Wu et al., 2017). The human/pig fetuses survived only 28 day, and it was not possible to observe whether the chimeric human ESCs could develop into mature functional cells in pigs for ethical issues. In this study, we confirmed that monkey/pig chimeras can form functional hepatocytes and renal cells in neonatal pigs. These functional cells could be isolated for further research and future clinical application.

Interspecies chimerism has been most successful in rodents, and these studies typically achieve a high proportion of chimerism and generate xenogeneic organs (Kobayashi et al., 2010; Isotani et al., 2011). The pluripotency levels of rodent PSCs are the highest known, as they are genuine naïve cells, and they can produce live offspring by tetraploid complementation, which is the most stringent test of pluripotency (Zhao et al., 2009; Li et al., 2017). Recent studies on xenogeneic chimerism in mice and rats showed that the pluripotency of rat ESCs affected kidney organ reconstruction in mice (Goto et al., 2019), demonstrating that the pluripotency of the donor cells influenced xenogeneic chimerism. Primate ESCs exist in a different phase of pluripotency than rodent ESCs, as they are primed rather than naïve (Brons et al., 2007; Nichols and Smith, 2009; Boroviak et al., 2014). The ratio of cmESCs in allogenic chimeras is only 0.1%–4.5% (Chen et al., 2015; Kang et al., 2018), which is significantly lower than with rodents. However, in previous reports, naïve hPSCs have lacked the competency to integrate into the mouse embryo (Gafni et al., 2013; Theunissen et al., 2014). In our study, chimeras were not obtained when the PSCs were transferred into naïve state or with overexpression of an anti-apoptotic gene. In agreement with Wu et al., we found that the PSCs cultured in the FAC condition showed a better chimeric contribution *in vitro*, and were more likely to generate normal individuals in the chimeric processes (Wu et al., 2017). Overall interspecies chimerism is complex, and in addition to the pluripotency of donor cells, its success involves several other aspects that facilitate interactions between the transplanted cells and recipient embryos. In our domestication system, the cell and embryo culture media were mixed in an optimal ratio, increasing the anti-apoptotic ability of cmESCs and improving the development of pig embryos, and allowing the successful generation of chimeric animals. Improvement of the culture conditions also played an important role in a previous homozygous chimera study (Kang et al., 2018). The proportion of chimerism in our study was 10–100 times higher than in the previously reported human/pig chimera (Wu et al., 2017). Therefore, optimizing the chimeric system is an effective method to increase the chimerism ratio.

Interspecies chimerism still has a long way to go before clinical application is possible. One obstacle is the need to increase the efficiency of homozygous chimerism by improving the pluripotency of cmESCs. There have been many studies addressing the acquisition of pluripotent and totipotent stem cells (Yang et al., 2017; Wang et al., 2018a). Second, information is needed regarding the molecular mechanisms underpinning the evolutionary differences that affect the efficiency of xenogeneic chimera development. Previous studies have reported successful chimeras containing human tissue cells, demonstrated by liver (Azuma et al., 2007), neural stem cell (Cohen et al., 2016), and hematopoietic immune system (Goyama et al., 2015) humanization, indicating that there is no obstacle to the remodeling of xenogeneic organs in human/mouse and human/pig chimeras. Here, we have used monkey cells to explore the potential of reconstructing chimeric human organs in a large animal model. We believe this work will facilitate the development of xenogeneic organogenesis by providing a better understanding of the processes of xenogeneic recognition, fate determination, and the proliferation and differentiation of primate stem cells during porcine development. The findings could pave the way toward overcoming the obstacles in the re-engineering of heterogeneous organs and achieve the ultimate goal of human organ reconstruction in a large animal.

## Material and methods

### Ethical approval

All animal experiments were performed in compliance with the guidelines of the Institute of Zoology, Chinese Academy of Sciences. The cross-species chimeric experiments were reviewed and approved by the ethics committee of the Institute of Zoology, Chinese Academy of Sciences.

### Cell culture

The cmESCs were a gift from Prof. Li, which were established as described previously (Chen et al., 2015). In brief, primed cmESCs were digested into clusters using ethylenediaminetetraacetic acid (EDTA), reseeded on feeder cells in primed medium (PM), and infected with simian immunodeficiency virus, harboring the sequence encoding EGFP driven by a cytomegalovirus (CMV)-enhanced chicken β-actin (CAG) promoter (Niu et al., 2010). After two passages, cmESCs sorted by fluorescence-activated cell sorting (FCAS) were detached using Accutase (Gibco, A11105-1) to obtain single cells, which were transferred to NHSM and FAC medium to generate naïve and intermediate cmESCs, respectively. Dome-shaped colonies were selected and transferred onto fresh feeder cells for further amplification. The D-ESCs were derived from cmESCs cultured in FM by mixing the FM with EM at a 1:1 ratio. Table S2 provides details about the culture media used.

### Pluripotency analysis in an *in vivo* teratoma assay

To induce teratoma formation, 1 × 10^6^ ESCs were subcutaneously injected into mice with severe combined immune deficiency. Teratomas were collected after approximately 4 week and fixed in 4% paraformaldehyde for paraffin embedding and hematoxylin and eosin staining according to standard procedures.

### Embryo micromanipulation and chimera assays

Procedures for porcine oocyte collection, *in vitro* maturation, IVF, PA production, somatic cell nuclear transfer (SCNT), and embryo culture were conducted as previously reported (Whitworth et al., 2014; Yuan et al., 2017). In brief, porcine ovaries were collected from a local slaughterhouse and transported to the laboratory within 1 h in normal saline at 37 °C. Oocytes were collected by aspiration and cultured for 42–44 h *in vitro*. Cumulus cells were then removed using 0.1% hyaluronidase, and matured oocytes at the MII stage were selected for IVF, PA, and SCNT. The embryos were cultured in porcine zygote medium in 5% carbon dioxide at 38.5 °C. In the *in vitro* experiment, 10 GFP-labeled ESCs were injected into porcine PA blastocysts collected 5 day after activation, followed by 48 h of culture. In the first 4 h, the injected embryos were cultured in ESC medium and then transferred to mixed ESC medium and EM at a 1:1 ratio for another 20 h, followed by culture in EM for the final 24 h. Porcine PA blastocysts were then collected for immunofluorescence analysis. In the *in vivo* experiment, 10–15 GFP-labeled ESCs were injected into IVF or NT blastocysts. After 24 h of *in vitro* culture, the injected embryos were transferred into recipient surrogates 5 day after estrus onset. Pregnancy was diagnosed by transabdominal ultrasonography using B-ultrasound (Model LX8000, Kangkaijie Instruments, China) 25–30 day later and confirmed every 2 week. Piglets were delivered naturally 114–117 day later.

### Immunofluorescence

The samples of cells, embryos (25–30 day), and tissue from neonatal pigs were fixed for 30 min in 4% paraformaldehyde in phosphate-buffered saline (PBS; 0.01 mol/L, pH 7.4) at room temperature (RT) and permeabilized with 0.5% Triton X-100 in PBS for 30 min at RT. The samples were then blocked in 1% bovine serum albumin in PBS for 1 h at RT after three 5 min washes in washing solution (0.1% Tween-20, 0.01% Triton X-100 in PBS), followed by incubation with primary antibodies (Table S3) overnight at 4 °C. After three 5 min washes in washing solution, cells or embryos were incubated with Alexa Fluor series fluorescent tag-conjugated secondary antibodies diluted in washing solution for 1 h at RT. After three washes with washing solution, nuclei were stained with 4’,6-diamidino-2-phenylindole (DAPI; 10 mg/mL in PBS) for 10 min. Immediately after three washes with washing solution, cells or embryos in microdroplets in a dish were imaged using a laser scanning inverted confocal microscope (LSM 780, Zeiss).

### Genomic PCR

Genomic PCR was performed to detect monkey cells in the porcine fetuses and tissues from neonatal pigs. The genomic DNA of pig fetuses was extracted from the formalin-fixed, paraffin-embedded (FFPE) slices using TIANamp Micro DNA Kit (TIANGEN, DP316). The genomic DNA of tissue from neonatal pigs was extracted using TIANamp Genomic DNA Kit (TIANGEN, DP304). PCR was performed using PrimeSTAR GXL DNA polymerase (Takara, R051A) followed by Sanger sequencing. Primer sequences used for PCR are provided in Table S4.

### Quantitative PCR (qPCR) analysis of monkey mtDNA

Analysis of monkey mtDNA was performed using SYBR Green Real-time PCR Master Mix (Takara, RR430A) on an Agilent Mx3005P qPCR system. Total DNA was isolated from porcine fetuses and neonatal porcine tissues. A monkey mtDNA control and a series of monkey-pig cell dilutions were run in parallel to estimate the degree of monkey cell contribution in interspecies chimeras. For normalization, an identical set of reactions was prepared using primers specific for an ultra-conserved non-coding element. Primer sequences used for genomic qPCR are listed in Table S4.

### RNA-seq library preparation and data processing

The cmESCs were cultured in PM, FM, and DM. Porcine PA blastocysts were collected 5 day after activation and cultured in PM, FM, and DM for 48 h. The cmESCs and porcine PA blastocysts were then collected for RNA-seq analysis. Total RNA was extracted from cultured cells using TRIzol reagent (Invitrogen, 15596026). For RNA-seq library construction, PolyA+ tailed RNA purification was performed for each sample using an E.Z.N.A. MicroElute RNA Clean-Up Kit (Omega Bio-tek). Sequencing was performed on an Illumina HiSeq 4000 sequencer with 150-bp paired-end sequencing reactions. RNA-seq data on monkey muscle samples downloaded from the GEO database (GSE102830) (Zhang et al., 2018) were used as a control. All RNA-seq reads were mapped to the *M*. *fascicularis* genome assembly (macFas5) using HISAT2 software (Kim et al., 2015), using annotated gene structures as templates and default parameters. Reads with unique genome locations were reserved for gene expression calculations in Cufflinks (version 2.0.2) using the option “–GTF”. Heat maps and cluster analysis were produced using the heatmap.2 and hcluster functions of R, respectively. The accession number for the RNA-seq expression values reported in this study is GSE.

### TUNEL assay

TUNEL assays were performed as previously described (Jachowicz et al., 2017). Embryos were collected 0 or 24 h after ESC injection and permeabilized in extraction buffer (50 mmol/L NaCl, 3 mmol/L MgCl_2_, 0.5% Triton X-100, and 300 mmol/L sucrose in 25 mmol/L HEPES, pH 7.4) for 5 min on ice. Next, the embryos were washed twice in extraction buffer without Triton X-100 and incubated with 1 U/mL of DNase I (Ambion, AM2222) in the same buffer for 5 min at 37 °C. After fixation, TUNEL assays were performed using a Click-iT TUNEL Alexa Fluor Imaging Assay Kit (Life Technologies) according to the manufacturer’s instructions. Immunofluorescence was performed as described above.

### Statistical analysis

Statistical analyses were performed using GraphPad Prism Software (GraphPad Prism 7.00). Each experiment included at least three independent biological replicates. For statistical comparison, Student’s *t*-test and one-way analysis of variance were employed. All data are presented as the mean ± standard error of the mean (SEM). *P < 0.05; **P < 0.01. Statistical parameters for specific experiments, including statistical analysis, statistical significance, and *n* values, are reported in the figure legends.

## Electronic supplementary material

Below is the link to the electronic supplementary material.

Supplementary material 1 (PDF 68509 kb)

## Abbreviations

BCL2L1: BCL2 like 1
cmESCs: cynomolgus monkey ESCs
D-ESCs: domesticated cmESCs
DM: domestic medium
EM: embryonic medium
EpiSCs: epiblast stem cells
ESCs: embryonic stem cells
FM: FAC medium
FOXA2: forkhead box A2
HNF4A: hepatocyte nuclear factor 4 alpha
ICM: inner cell mass
IVF: *in vitro* fertilization
NT: nuclear transfer
Pdx1: pancreatic and duodenal homeobox 1
POU5F1: POU class 5 homeobox 1
PRDM14: PR/SET domain 14
PSCs: pluripotent stem cells
SALL1: spalt like transcription factor 1
SOX1: SRY-box transcription factor 1
SOX2: SRY-box transcription factor 2
TBX6: T-box transcription factor 6
