# Supplementary material for: Domesticated cynomolgus monkey embryonic stem cells allow the generation of neonatal interspecies chimeric pigs
Source: Protein Cell. 2019 Nov 28;11(2):97–107. doi: 10.1007/s13238-019-00676-8 (PMC6954905; doi:10.1007/s13238-019-00676-8)
Supplement: Supplementary file 1 — Supplementary material 1 (PDF 68509 kb) [file 13238_2019_676_MOESM1_ESM.pdf]

Supplementary Information

Table S1. Developmental efficiencies of cmESCs at different pluripotent state and over expression BCL2L1 after chimeric operation.
Table S2. The information of culture medium.
Table S3. Antibodies information are listed.
Table S4. PCR primers for genomic PCR and qPCR analysis of monkey mtDNA
Figure legends for figure S 1-3

Table S1. Developmental efficiencies of cmESCs at different pluripotent state and over expression BCL2L1 after chimeric operation.

cmESC lines	Sex	State	Stage of injection	NO. embryo transferred	NO. recipient	NO. development in vivo (Full-term)
cmESC-1	Male	Primed	Blastocyst	517	5	0
		Intermediate	Blastocyst	286	3	0
		Naive	Blastocyst	400	4	0
BCL2L1	Male	Primed	8 - cell	218	2	0
			Blastocyst	425	4	0

Table S2. The information of culture medium

Primed medium	50 ml	Source	Identifier
DMEM/F12	41.5 ml	Gibco	10565-018
15 % KOSR	15 %	Gibco	12618013
NEAA	1 %	Gibco	11140-050
Glumax	1 mM	Gibco	35050-061
Mercaptoethanol	0.1 mM	Gibco	21985-023
Basic FGF	10 ng/ml	R&D	233-FB
FAC medium	50 ml	Source	Identifier
DMEM/F12	23.5 ml	Gibco	10565-018
Neurobasl	23.5 ml	Gibco	21103-049
N2	0.5 %	Gibco	17502-048
B27	1 %	Gibco	17504-044
Glumax	1 mM	Gibco	35050-061
NEAA	1 %	Gibco	11140-050
mercaptoethanol	0.1 mM	Gibco	21985-023
bFGF	12 ng/ml	R&D	233-FB
ActivinA	50 ng/ml	R&D	338-AC
CHIR99021	3 μM	Selleckchem	S2924
NHSM	50 ml	Source	Identifier
KO-DMEM	48 ml	Gibco	10829-018
Albumaxl	0.5 g	Gibco	11020-021

Glumax	1 mM	Gibco	35050-061
NEAA	1 %	Gibco	11140-050
B-mer	0.1 mM	Gibco	21985-023
N2	0.5 ml	Gibco	17502-048
humam insulin	12.5 μg/ml	Roche	11376497001
CHIR99021	3 μM	Selleckchem	S2924
PD0325901	1	Stemgent	04-0006
SP600125	5 μM	TOCRIS	1496
SB203580	5 μM	TOCRIS	1202
human Lif	20 ng/ml	PeproTech	AF-300-05-100UG
bFGF	8 ng/ml	R&D	233-FB
VC	50 μg/ml	Sigma	A4403
TGB1	1 ng/ml	PeproTech	100-21
Embryonic medium	50 ml	Source	Identifier
L-Glutamine	0.0073 g	Sigma	G8540-25G
Hypotaurine	0.0273 g	Sigma	H1384-1G
Gentamicin sulfate salt	0.0025 g	Sigma	G1264-5G
MEM NON-ESSENTIAL AMION ACID SOLUTIDN 100x	500 μl	Sigma	M7145
BME AMINO ACIDS SOLUTICN 50x	1 ml	Sigma	B6766
BSA	0.15 g	Sigma	A6003-25G
Nacl	0.3156 g	Sigma	S-5886
NaHCO3	0.1053 g	Sigma	S-4019
Kcl	0.0373 g	Sigma	P-5405
KH2PO4	0.0024 g	Sigma	P-5655
MgSO4	0.0024 g	Sigma	M-2643
Ca-lactate-5H2O	0.0308 g	Sigma	C-8356
Na-pyvate	0.0011 g	Sigma	P-3662
Myo-Inositol	0.0250 g	Sigma	I-7508
Phenol Red	0.0005 g	Sigma	P-5530
Sigma H2O	48.5 ml	Sigma	W-1503

Table S3. Antibodies information are listed.

Antibodies	Source	Identifier
Goat polyclonal Anti-Sox2(Y-17)	Santa Cruz	Cat#SC-17320
Mouse Monoclonal Anti-OCT3/4	Santa Cruz	Cat#SC-5279
Rabbit polyclonal Anti-Nanog	Abcam	Cat#Ab80892
Mouse Anti-hNA	Millipore	Cat#MAB1281
Anti-GFP	Abcam	Cat#Ab290
Human Anti-FoxA2	R&D	Cat#AF2400
Human Anti-TBX6	R&D	Cat#AF4744

Human Polyclonal Anti-SOX1	R&D	Cat#AF3369
Anti-SALL1	Santa Cruz	Cat#SC-46037
Mouse monoclonal Anti-HNF-4-alpha	Abcam	Cat#Ab41898
Alexa Fluor 488 donkey anti-Mouse Ig （H+L）	Invitrogen	REF:A21202 LOT:1890861
Alexa Fluor 488 donkey anti-Rabbit Ig （H+L）	Invitrogen	REF:A21206 LOT:1874771
Alexa Fluor 488 donkey anti-Goat Ig （H+L）	Invitrogen	REF:A11055 LOT:1869589
Alexa Fluor 594 donkey anti-Mouse Ig （H+L）	Invitrogen	REF:A21203 LOT:1820027
Alexa Fluor 594 donkey anti-Rabbit Ig （H+L）	Invitrogen	REF:A21207 LOT:1890862
Alexa Fluor 594 donkey anti-Goat Ig （H+L）	Invitrogen	REF:A11058 LOT:1842799

Table S4. PCR primers for genomic PCR and qPCR analysis of monkey mtDNA

Name	Sequence(5’-3’)
CSF-R	AACCTGAGTCTGCCAAGGACTAGC
CSF-F	TTCCACACACCACTGGCCATCTTC
monkey-mit-178F	CATATCACCATTGTATTAACGGGCCTT
monkey-mit-178R	TAGAAGGATAATTGGAGCGAGGTGT
SOX2-F	TTTGTCGGAGACGGAGAAGC
SOX2-R	CCTGCATCATGCTGTAGCTG
UCNE-F	AACAATGGGTTCAGCTGCTT
UCNE-R	CCCAGGCGTATTTTTGTTCT

Figure S1. Chimeric competency of cmESCs in different pluripotent states.

(A) Immunofluorescence images of cmESCs with different pluripotent states stained with POU5F1. Scale bar, 100 μm. (B) Representative florescence and embryoid bodies images showing the morphologies of cmESCs with different pluripotent states. Scale bar, 200 μm. (C) Representative images of three embryonic germ layers of teratomas generated by injection of cmESCs with different pluripotent states into SCID mouse. Scale bar, 100 mm. (D) The karyotypes of cmESCs with different pluripotent states. (E) Representative images of porcine PA embryos showing integrated GFP+ cmESCs with different pluripotent states in cultured embryos. Scale bars, 200 μm. (F) The percentage of GFP positive embryos in total embryos.

Figure S2. Chimeric competency of cmESCs with anti-apoptotic gene BCL2L1-overexpressing

(A) Representative images of porcine PA embryos showing integrated GFP positive cmESCs with anti-apoptotic gene BCL2L1 overexpressing. Scale bars, 50 μm. (B) The percentage of GFP positive embryos in total embryos. (C) Number of cmESCs with anti-apoptotic gene BCLX overexpressing that integrated into the porcine blastocyst. (D) The karyotypes of D-ESCs. (E) The quantification of POU5F1 and SOX2-positive cells in D-ESCs. (F) Representative images of three embryonic germ layers of teratomas generated by injection of D-ESCs into SCID mouse. Scale bar, 100 mm.**p < 0.01, student's *t* test. Error bars represent mean ± SEM (n=5).

Figure S3. Identify of the chimeric situation in the neonatal pig tissue. (A) Representative gel images showing genomic PCR analyses of neonatal porcine tissue derived from blastocyst injection of D-ESCs using cynomolgus monkey-specific CSF and Mit primer. A pig-specific primer, SOX2, was used as the loading control. CM, cmESCs. Mix, pig / cmESC (1:1). NC, negative control without genomic DNA loaded. (B) Representative immunofluorescence images of GFP-labeled D-ESCs in the chimeric neonatal pig of testis, epididymis, ovary and brain. Scale bar, 100 μm.

Figure S1

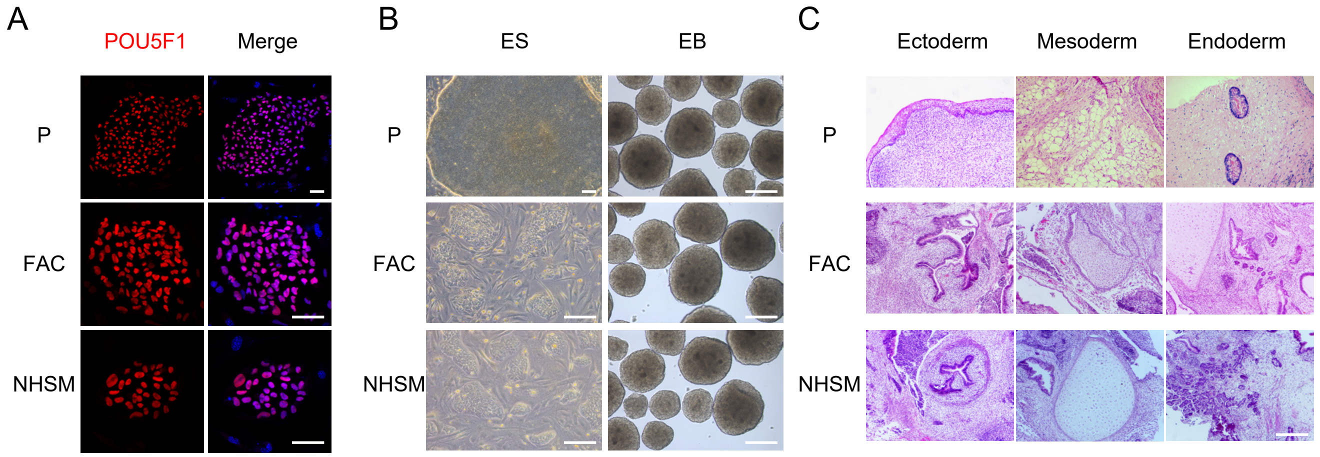
A
POU5F1
Merge
P
FAC
NHSM
B
ES
EB
P
FAC
NHSM
C
Ectoderm
Mesoderm
Endoderm
P
FAC
NHSM

Figure S1

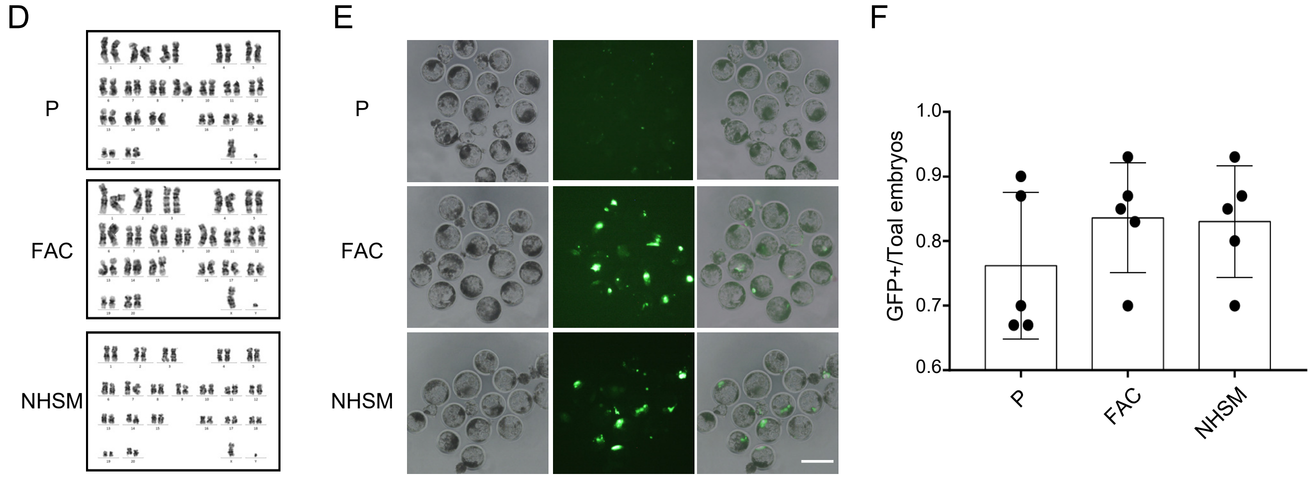

Figure S2

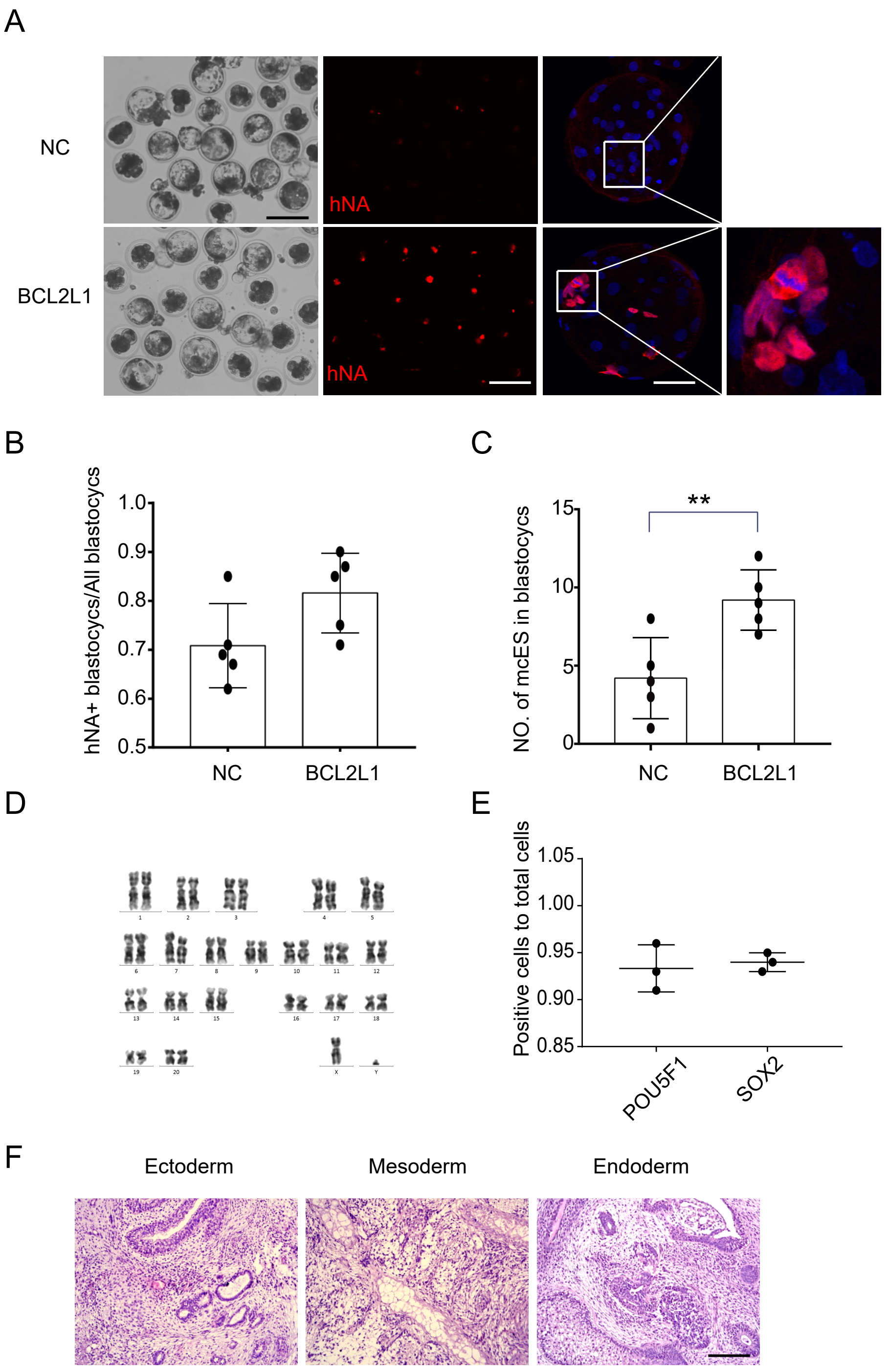
A
NC
BCL2L1
hNA
hNA
B
hNA+ blastocycs/All blastocycs
1.0
0.9
0.8
0.7
0.6
0.5
NC
BCL2L1
C
NO. of mcES in blastocycs
15
10
5
0
**
NC
BCL2L1
D
E
Positive cells to total cells
1.05
1.00
0.95
0.90
0.85
POU5F1
SOX2
F
Ectoderm
Mesoderm
Endoderm

Figure S3

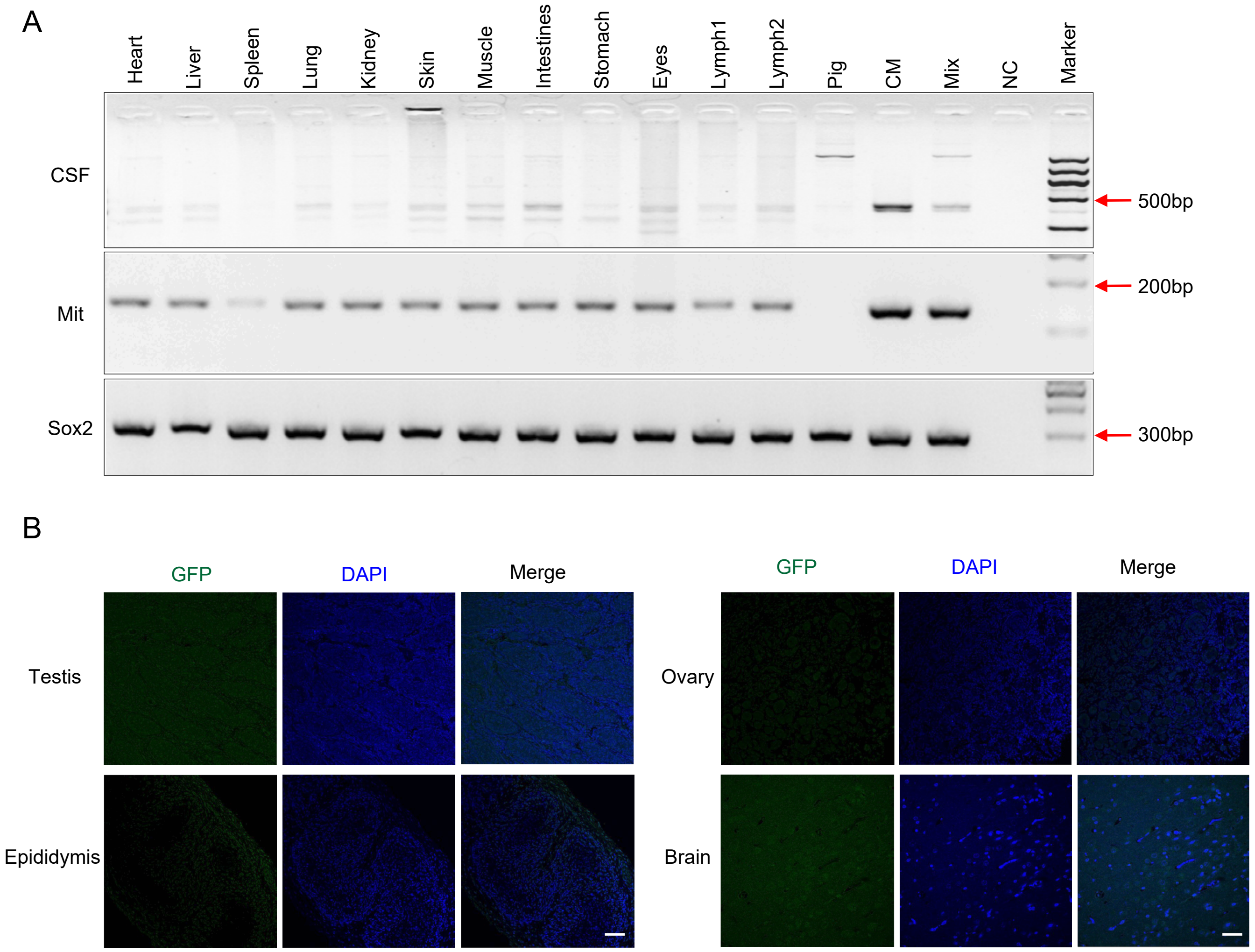
A
Heart
Liver
Spleen
Lung
Kidney
Skin
Muscle
Intestines
Stomach
Eyes
Lymph1
Lymph2
Pig
CM
Mix
NC
Marker
CSF
500bp
Mit
200bp
Sox2
300bp
B
GFP
DAPI
Merge
Testis
Epididymis
GFP
DAPI
Merge
Ovary
Brain
